# Bacteremia in Children Hospitalized with Respiratory Syncytial Virus Infection

**DOI:** 10.1371/journal.pone.0146599

**Published:** 2016-02-12

**Authors:** Miriam Cebey-López, Jacobo Pardo-Seco, Alberto Gómez-Carballa, Nazareth Martinón-Torres, José María Martinón-Sánchez, Antonio Justicia-Grande, Irene Rivero-Calle, Elli Pinnock, Antonio Salas, Colin Fink, Federico Martinón-Torres, GENDRES network

**Affiliations:** 1 Grupo de Investigación en Genética, Vacunas, Infecciones y Pediatría (GENVIP - www.genvip.org), Hospital Clínico Universitario and Universidade de Santiago de Compostela (USC), Galicia, Spain; 2 Translational Pediatrics and Infectious Diseases Section, Department of Pediatrics, Hospital Clínico Universitario de Santiago, Santiago de Compostela, Galicia, Spain; 3 Unidade de Xenética, Departamento de Anatomía Patolóxica e Ciencias Forenses, and Instituto de Ciencias Forenses, Grupo de Medicina Xenómica (GMX), Facultade de Medicina, Universidade de Santiago de Compostela, Galicia, Spain; 4 Micropathology Ltd., University of Warwick Science Park, Coventry, United Kingdom; University Hospital San Giovanni Battista di Torino, ITALY

## Abstract

**Background:**

The risk of bacteremia is considered low in children with acute bronchiolitis. However the rate of occult bacteremia in infants with RSV infection is not well established. The aim was to determine the actual rate and predictive factors of bacteremia in children admitted to hospital due to confirmed RSV acute respiratory illness (ARI), using both conventional culture and molecular techniques.

**Methods:**

A prospective multicenter study (GENDRES-network) was conducted between 2011–2013 in children under the age of two admitted to hospital because of an ARI. Among those RSV-positive, bacterial presence in blood was assessed using PCR for *Meningococcus*, *Streptococcus pneumoniae*, *Haemophilus influenzae*, *Streptococcus pyogenes*, *Klebsiella pneumoniae*, *Pseudomonas aeruginosa*, *Escherichia coli*, and *Staphylococcus aureus*, in addition to conventional cultures.

**Results:**

66 children with positive RSV respiratory illness were included. In 10.6% patients, bacterial presence was detected: *H*. *influenzae* (*n* = 4) and *S*. *pneumoniae* (*n* = 2). In those patients with bacteremia, there was a previous suspicion of bacterial superinfection and had received empirical antibiotic treatment 6 out of 7 (85.7%) patients. There were significant differences in terms of severity between children with positive bacterial PCR and those with negative results: PICU admission (100% vs. 50%, *P-*value = 0.015); respiratory support necessity (100% vs. 18.6%, *P-*value < 0.001); Wood-Downes score (mean = 8.7 vs. 4.8 points, *P*-value < 0.001); GENVIP scale (mean = 17 vs. 10.1, *P-*value < 0.001); and length of hospitalization (mean = 12.1 vs. 7.5 days, *P-*value = 0.007).

**Conclusion:**

Bacteremia is not frequent in infants hospitalized with RSV respiratory infection, however, it should be considered in the most severe cases.

## Introduction

Respiratory syncytial virus (RSV) is the main cause of viral acute respiratory infection and leads to hospitalization in infants and young children worldwide, being responsible for 60% to 80% of hospitalizations for bronchiolitis [1]. The main complication of viral respiratory infections is bacterial co-infection and respiratory failure. The synergism between virus and bacteria has been widely discussed in the literature, particularly for respiratory viruses and secondary bacterial pneumonia [2]. Bacteremia rates reported in children with respiratory illness are low, with rates <1.2% in RSV—confirmed cases [3–7]. In those requiring mechanical ventilation, bacterial co-infection rates in respiratory samples are consistently higher but may vary, ranging from 21.0% to 43.9% [8,9]. However, the prevalence, clinical phenotype and course of bacteremia with pathogenic bacteria in the setting of acute RSV infection in infants with no other risk factors, are questions that have not been investigated in sufficient detail, even less with the use of molecular diagnostics.

The aim of this study was to determine the actual rate and predictive factors of bacteremia in children admitted to hospital due to confirmed RSV acute respiratory illness using both conventional culture and molecular techniques.

## Materials and Methods

### Study design and recruitment criteria

This is an observational prospective sub-study performed from January 2011 to January 2013, in the framework of GENDRES (Genetic, Vitamin D and Respiratory Infections Research Network– www.gendres.org), a research network on pediatric acute respiratory infections that includes 13 Spanish tertiary hospitals. For this study, previously healthy infants under the age of two admitted to any of the participant hospitals in the GENDRES network with confirmed RSV infection were included. Patients with a history of known immunodeficiency, severe cardiac anomaly, severe chronic lung disease, neuromuscular disease, chromosomal abnormalities or any ongoing chronic disease were excluded from the study. A complete clinical record, a blood sample and a nasopharyngeal sample were collected in all included participants.

The severity of each respiratory episode was ranked as follows: 1) physician criteria (mild, moderate or severe); 2) Wood-Downes scale (0 to 10 points; mild < 3, moderate 4–7, severe > 8); and 3) a newly developed scale -named GENVIP score- (0 to 20 points) that assesses food tolerance, degree of medical intervention needed, respiratory distress, respiratory frequency, apnea, malaise and fever (article in preparation; data in S1 Table [10]).

The study was performed according to Good Epidemiological Practice and written informed consent was obtained from a parent or legal guardian for each subject before study inclusion. The study was approved by the Ethics Committee of Clinical Investigation of Galicia (CEIC ref 2010/015).

### Clinical data collection and laboratory methods

The patients’ clinical and demographic data were collected using a secure web-based platform. RSV status was determined both by conventional methods/rapid test in the referring hospital, and by molecular techniques in nasopharyngeal secretions. For RSV assessment by molecular techniques, a nasopharyngeal sample was obtained for the study during hospitalization, using either a sterile feeding tube and injector for nasopharyngeal aspirate/wash, or a sterile nylon swab (FLOQSwabsTM by Copan Diagnostics, Brescia, Italy), without culture medium. A panel of 19 viruses including RSV was sought by nested polymerase chain reaction (PCR). Methods were previously described in Cebey-López et al. [11].

A peripheral blood sample for analysis was also collected from each patient at the same time as the swab, in an EDTA *BD Vacutainer® K3E 5*.*4 mg* tube (Becton Dickinson, Franklin Lakes, Plymouth, UK). In some patients, the initial conventional bacterial test was performed on hospital admission samples, whilst PCR was performed using samples obtained after the patients were transferred to PICU and recruited into the study. PCR results were not shared with clinicians due to all PCR were performed simultaneously off-patient. Blood samples were pretreated as follows: 250ul of blood was mixed with 25ul lysozyme (100 mg/ml, Sigma-Aldrich, St Louis, Missouri, USA) and 10ul lysostaphin (1mg/ml, Sigma-Aldrich, St Louis, Missouri, USA), vortexed briefly and incubated at 37°C for 30 minutes. 0.1g lysing matrix B beads (MP Biomedicals, Santa Ana, California, USA) were added and the mixture was agitated at 30Hz for 2x30s using a TissueLyser II (Qiagen, Hilden, Germany). 200ul lysis buffer AL (Qiagen, Hilden, Germany) was added and the samples were centrifuged at 5,500 rpm for 2 minutes to pellet the lysis beads. 200ul of the lysed blood sample was then transferred onto a Biorobot MDx (Qiagen, Hilden, Germany) and total nucleic acid extracted using silica membrane chemistry and eluted in 200ul of elution buffer AE (QIAamp DNA Blood Biorobot MDx extraction kit, Qiagen, Hilden, Germany).

Nested PCR was then performed to detect the presence of ten different organisms, as below (Table 1).

**Table 1:** Target genes and organisms detected by nested PCR.

Organism	Target gene	Amplicon size (bp)
Neisseria meningitides	*porA*	947
Streptococcus pneumoniae	*ply*	209
Haemophilus influenzae	*P6OMP*	353
Group A Streptococcus	*mf*	419
Group B Streptococcus	*cfb*	154
Acinobacter baumanii	*gltA*	155
Klebsiella pneumoniae	*mdh*	193
Pseudomonas aeruginosa	*oprL*	163
Escherichia coli	*phoA*	196
Staphylococcus aureus	*femA*	95

The PCRs were performed as two screens, with the first round reactions multiplexed for targets 1–5 and 6–10 respectively. All first round reactions were performed in 50ul reaction volumes consisting of 20ul template (nucleic acid extracted from blood as described above) and 30ul mastermix (MyTaqHS, Bioline Reagents, London, UK) with each primer added to a final reaction concentration of 0.2uM. Thermal cycling conditions for the first round reactions were: 95°C for 2 minutes followed by 30 cycles of 95°C for 15s, 55°C for 15s, 72°C for 15s; this was performed using a TRIO block thermocycler (Biometra Gmbh, Göttingen, Germany). 1ul of first round template was transferred into 24ul of second round mastermix (MyTaqHS mastermix, Bioline Reagents, London, UK) containing second round primers at a final reaction concentration of 0.2uM for each primer and 1ul of Evagreen (Biotium, Hayward, California, USA) per reaction. Second round reactions were performed on a Lightcycler 480 (Hoffman-La Roche, Basel, Switzerland) using the following cycling conditions: 95°C for 2 minutes followed by 30 cycles of 95°C for 20s, 55°C for 20s, 76°C for 20s with fluorescence acquisition at 76°C. This was followed by a melt curve in which fluorescence was acquired from 75–95°C and melt curve analysis used to detect specific amplification.

DNA extraction and PCR amplification of bacterial targets was performed by a commercial referral laboratory, Micropathology Ltd (MCP). MCP is accredited by Clinical Pathology Accreditation (UK) Ltd (CPA, a subsidiary of UKAS) to provide diagnostic pathology services in the UK. All molecular PCR assays are validated to CPA standards. This process includes primer specificity checking by an in-house method based on analysis of publically available sequence data to ensure that observed variation within each species is accounted for and that no cross-reactivity to other targets can be identified. The sensitivity of the PCRs for all ten targets is <5 gene copies/reaction. This was derived using a combination of quantitated PCR control material (AmpliRun, Vircell, Granada, Spain) and synthetic copies of the target amplicon, quantitated using an automated electrophoresis platform (MultiNA, Shimadzu, Kyoto, Japan).

Bacteremia was defined as the positivity of PCR and/or blood culture. Bacterial superinfection diagnosis relied on the discretion of the recruiting physician according to clinical data, inflammatory markers, radiological findings and/or appropriate cultures. PCR was done after children discharge so results were not shared with clinicians. All PCR were performed simultaneously off-patient. Respiratory support was considered as either invasive (mechanical ventilation) or non-invasive ventilation (CPAP, BiPAP). The severity of each respiratory episode was ranked by physician criteria (mild, moderate or severe). The respiratory distress was rated in the worst moment of the illness. Spanish national immunization program includes Hib vaccine but not pneumococcal conjugate vaccine. However average vaccination coverage with pneumococcal conjuagate vaccine is estimated to be 60–65% through the private market.

### Data analysis

We performed all the analyses using R Software, version 3.0.2 (http://www.r-project.org). General data are presented as percentages or means with 95% confidence intervals. Different statistical tests were used to assess the association between the variables: Fisher’s exact test for discrete variables and Wilcoxon test for continuous variables.

## Results

Out of 130 eligible patients, 66 were RSV positive (data in S1 Fig). 92.4% of these (*n* = 61) were <12 months of age and the majority (66.7%) of the patients were boys. All children were correctly vaccinated according to the recommended national immunization schedule. Diagnosis at discharge was predominantly bronchiolitis 78.8% (*n* = 54) and pneumonia 6.1% (*n* = 4)

**Fig 1:**
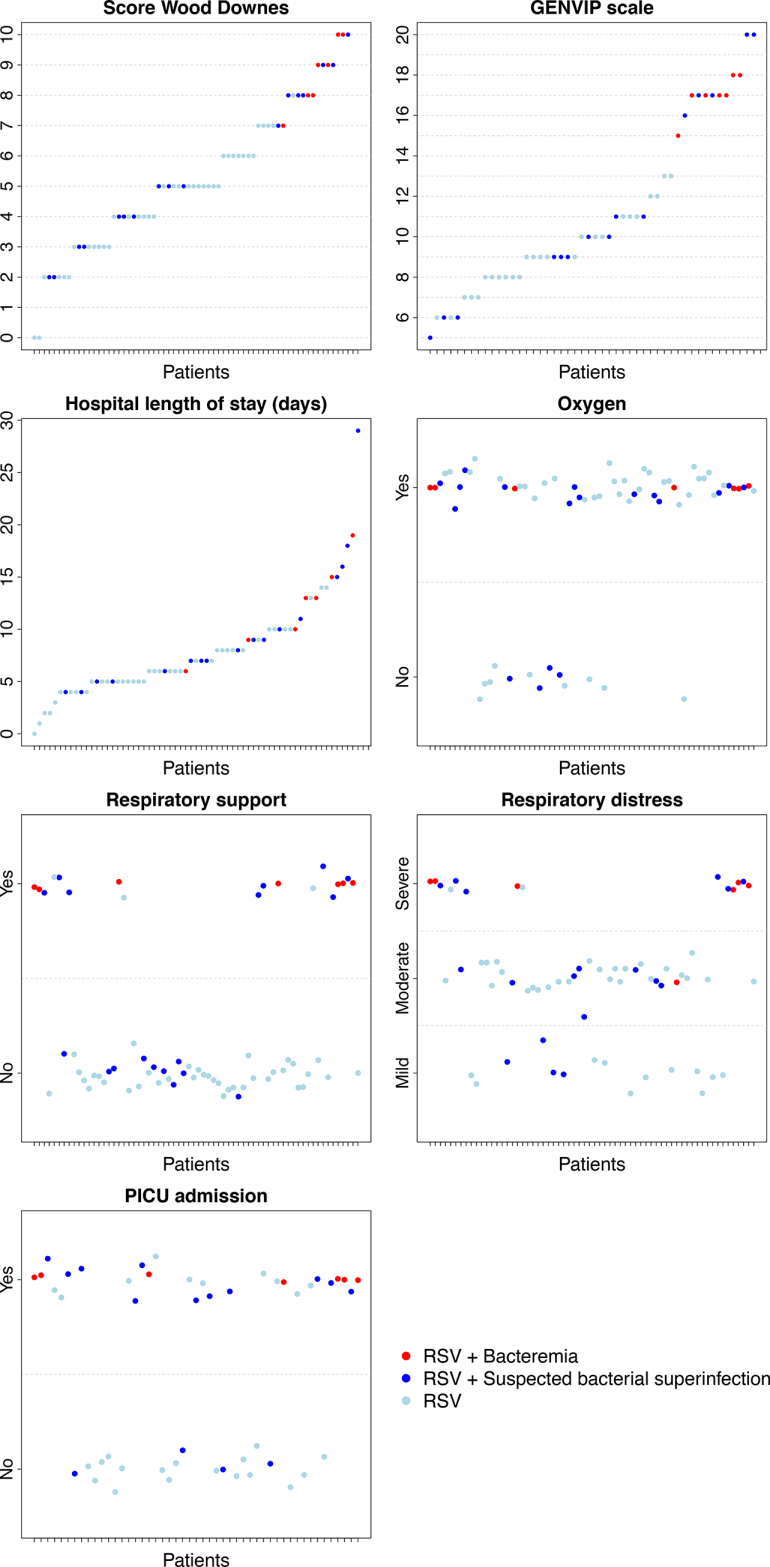
Severity parameters for the patients: Wood Downes score, GENVIP score, length of hospitalization, oxygen, respiratory support, respiratory distress and PICU admission. Patients are classified as: positive RSV in nasopharyngeal sample, positive RSV with confirmed bacteremia, and positive RSV and suspected bacterial superinfection.

34.9% (*n* = 23) of the RSV positive patients had a suspected bacterial superinfection according to the referring physician. Molecular assessment revealed bacterial presence in the blood of seven (10.6%) patients (Table 2). No differences were found between those with negative or positive PCR in terms of sex distribution, age, family history, vaccination rate, diagnosis or suspicion of bacterial superinfection. There were significant differences in terms of illness severity between children with positive bacterial PCR and those with negative results: PICU admission (100% vs. 50%, *P*-value = 0.015) and respiratory support necessity (100% vs. 18.6%, *P*-value < 0.001). Patients with confirmed bacteremia had a more severe respiratory affection than those with no bacteria identified in blood (Table 2). Both the Wood-Downes score and the GENVIP scale indicated a worse value in the blood PCR positive patients (mean = 8.7 points and 17.0 points, respectively) than in the blood PCR negative patients (mean = 4.8 points and 10.1 points) (*P*-value < 0.001 for both). Hospitalization was longer for children with PCR-confirmed bacteremia (mean = 12.1 vs. 7.5 days, *P*-value = 0.007) (Fig 1).

**Table 2:** Summary of the characteristics of RSV cohort and comparison between those with positive and negative blood PCR for bacteria. General data are presented as percentages or means with 95% confidence intervals. Different statistical models were used to assess the association between the variables: Fisher’s exact test (1) for discrete variables and Wilcoxon test (2) for continuous variables.

Variables	Total cohort (*n* = 66)	Negative PCR (*n* = 59)	Positive PCR (*n* = 7)	*P-value*
**Demographics**				
Sex. Female^1^	33.3% (22/66)	35.6% (21/59)	14.3% (1/7)	0.409
Age (months)^1^				1.000
< 12	92.4 (61/66)	91.5 (54/59)	100 (7/7)	
12–24	3.0% (2/66)	3.4% (2/59)	0.0% (0/7)	
> 24	4.5% (3/66)	5.1% (3/59)	0.0% (0/7)	
**Family history**				
Asthma^1^	30.3% (20/66)	71.2% (42/59)	57.1% (4/7)	0.425
Respiratory conditions^1^	30.3% (20/66)	28.8% (17/59)	42.9% (3/7)	0.425
**Medical history**				
Premature^1^	4.8% (3/63)	3.6% (2/56)	14.3% (1/7)	0.302
Pneumococcal vaccine	48.5 (32/66)	52.5% (31/59)	14.3% (1/7)	0.106
**Clinical data**				
Oxygen needed^1^	80.3% (53/66)	78.0% (46/59)	100.0% (7/7)	0.329
Any kind of respiratory support^1^	27.3% (18/66)	18.6% (11/59)	100.0% (7/7)	<0.001
Diagnosis^1^				0.739
Bronchiolitis	78.8% (52/66)	76.3% (45/59)	100.0% (7/7)	
Pneumonia	6.1% (4/66)	6.8% (4/59)	0.0% (0/7)	
Bronchial hyperreactivity	15.2% (10/66)	16.9% (10/59)	0.0% (0/7)	
Respiratory distress^1^				0.001
Mild	22.7% (15/66)	25.4% (15/59)	0.0% (0/7)	
Moderate	53.0% (35/66)	57.6% (34/59)	14.3% (1/7)	
Severe	21.2% (14/66)	13.6% (8/59)	85.7% (6/7)	
PICU admission^1^	57.1% (28/49)	50.0% (21/42)	100.0% (7/7)	0.015
Fever^1^				0.733
Low-grade fever (37–38°C)	37.5% (18/48)	36.6% (15/41)	42.9% (3/7)	
Fever (> 38°C)	50.0% (24/48)	48.8% (20/41)	57.1% (4/7)	
Wood Downes Score (mean-SD)^2^	5.2 (2.4)	4.8 (2.2)	8.7 (1.1)	<0.001
GENVIP scale (mean-SD)^2^	11.1 (4.1)	10.1 (3.6)	17.0 (1.0)	<0.001
Hospital length of stay (mean-SD)^2^	8.0 (4.8)	7.5 (4.7)	12.1 (4.3)	0.007
Suspected bacterial superinfection^1^	50.0% (33/66)	45.8% (27/59)	85.7% (6/7)	0.105
Blood culture				
Done. Yes^1^	60.0% (27/45)	55.3% (21/38)	85.7% (6/7)	0.215
Positive^1^	3.7% (1/27)	0.0% (0/21)	17.0% (1/6)	0.222

^1^Fisher’s exact test.

^2^Wilcoxon test

The pathogen found were *H*. *influenzae* (*n* = 4), *S*. *pneumoniae* (*n* = 1) and both simltaneously (*n* = 2). The cycle thresholds were <20 except for S. pneumoniae in the co-infection cases, in which the cycle threshold was >25. Only in one of these cases this bacteria had also been identified by conventional cultures.

In nasopharyngeal samples, more than one virus was detected in 35 out of 66 (53.0%) patients by molecular techniques. In bacterial PCR positive patients, viral co-infection was observed in 5 out of 7 (71.4%) subjects (Table 3). 30 patients with viral co-infection and negative PCR were identified (*n* = 24 patients with two virus and *n* = 6 with three virus). No statistical differences were observed in terms of viral co-infection between positive and negative bacterial PCR (*P*-value = 0.433)

**Table 3:** Description of RSV infected patients with positive blood bacterial PCR. Abbreviations: NINV: noninvasive ventilation; INV: invasive ventilation; RSV: respiratory syncytial virus; PCR: polymerase chain reaction.

Patient	Blood PCR -Bacteria	PCR—Virus	Respiratory distress	Wood-Downes Score	Oxygen	Respiratory support	Bronchiolitis diagnosis	Suspected bacterial superinfection
**1**	H. influenzae/ S. pneumoniae	RSV + Rhinovirus	Severe	9	Yes	NINV	Yes	Yes
**2**	H. influenzae	RSV	Severe	10	Yes	NINV	Yes	Yes
**3**	S. pneumoniae	RSV + Rhinovirus + Bocavirus	Severe	10	Yes	INV	Yes	Yes
**4**	H. influenzae	RSV + Bocavirus	Moderate	7	Yes	NINV	Yes	Yes
**5**	H. influenzae/ S. pneumoniae	RSV + Bocavirus	Severe	8	Yes	NINV	Yes	No
**6**	H. influenzae	RSV + Rhinovirus + Coronavirus	Severe	9	Yes	INV	Yes	Yes
**7**	H. influenzae	RSV	Severe	8	Yes	INV	Yes	Yes

Antibiotic administration in patients with suspected bacterial superinfection was recorded in 50.0% (*n* = 33) of the RSV-infected patients and in 3.3% (*n* = 1) of these patients a bacterial superinfection was confirmed by conventional blood cultures. In the patients with confirmed bacteremia by PCR, n = 6 patients were treated with antibiotics due to suspected bacterial superinfection. In these 6 patients, wide spectrum antibiotics were used.

There was no correlation between the patients suspected of superinfection and/or prescribed antibiotic by the referring physician, and those with positive blood PCR results (Cohen’s kappa coefficient bacterial superinfection-PCR = 0.15). A total of 87.5% of the patients presented fever: > 38°C in 50% of the included children (24 out of 48), and low-grade fever in 18 of them (37.5%). Fever frequency in children with bacteremia confirmed by PCR was similar to that in the rest of the cohort (*P*-value = 0.733).

## Discussion

One out of every ten previously healthy children hospitalized due to RSV had bacteremia, and these patients experienced a more severe disease. Conversely, half ot the remaining RSV patients included, received empirical antibiotic therapy despite the bacteremia work-up result was negative.

The prevalence of bacteremia in children with RSV infection reported in the literature is low, ranging between 0.6 and 1.1% [3–5,12–14]. Our study found rates of concurrent bacteremia ten times higher (10.6%). In the studies cited, only conventional cultures were performed, whereas molecular methods were applied in our series. RSV has been linked to seasonal increases in *S*. *pneumoniae* disease [15], as well as to other viruses such as influenza, but the underlying mechanisms between viral and bacterial synergism are complex and remain unclear. Immunization programs with conjugate vaccines against invasive *H*. *influenzae* serotype b and *Streptococcus pneumoniae* have changed the frequency of bacteremia in febrile infants (10,13). In our series, only 14.3% of the PCR positive patients had been vaccinated against pneumococcal disease, as compared to PCR negative ones, with 52.5% having received the vaccine. Animal studies found important mechanism of viral-bacterial synergy between bovine RSV and Histophilus somni and other bacterial pathogens [16]. This could indicate that RSV and H.influenzae have the same infection pattern in human patients.

The diagnosis of bacterial superinfection is most often made on clinical grounds, and not always confirmed microbiologically. Antibiotics should not be administered to RSV-infected children unless complications such as secondary bacterial illness occur [17,18]. In our series, blood cultures were not carried out systematically, but only when a bacterial superinfection was suspected by the referring physician. Blood culture is considered the gold standard for bacteria detection, but has a low sensitivity and some bacteria are difficult to culture. Blood PCR–much more sensitive–was performed in all the recruited patients but the clinical relevance of its positive result is not clear. Detecting circulating DNA in the blood by PCR has some limitations and it is essential to extrapolate always the results obtained to the clinical grounds to see if both PCR results and the clinical phenotype are consistent [19]. The quantitative PCR and the cycle threshold value are inversely correlated with the bacteria load and could be an indicator to avoid false positive results. In our cohort the cycle threshold was <20, but as we only have seven positive samples we could not establish maximum cutoff levels.

In our series, the physician suspected bacterial superinfection in half of the cases, a proportion similar to that in Thibeault et al.’s study [20]. Even though a blood culture was obtained for many of our patients (60.0%), only 3.8% (*n* = 1) yielded a positive result. Furthermore, we failed to find any correlation between PCR-confirmed bacteremia and clinical suspicion of bacterial superinfection (Cohen’s kappa coefficient = 0.15). Therefore, in agreement with other studies [14,21], we found that children with uncomplicated bacterial RSV infection are often overtreated with empirical antibiotics.

Empirical antibiotic treatment is often prescribed in practice to children hospitalized due to a confirmed viral ARI based on fever presence or persistence. Between 23 and 31% of cases of bronchiolitis are associated with fever [22], and in our series the fever rate (37 and 50%) was in the range previously described of 45 to 65% for children hospitalized with RSV [23]. However, the risk of bacteremia is low in febrile children with bronchiolitis [4,6,22], as corroborated in our series, where fever was not a predictor for bacteremia (*P*-value = 0.733).

Randolph et al. [9] studied the risk of bacterial infection in children infected with RSV admitted to PICU, and using blood culture they found a rate of bacteremia of only 0.6%. In our study, the rate of bacteremia found by PCR in children who required PICU admission is higher (25.0%), probably due to the higher sensitivity of molecular techniques. Bacteremic patients had a more severe course according to PICU admission rates, respiratory support necessity, clinical scales and length of hospitalization. Bloomfield et al. [14] suggest that empirical antibiotics should be considered for any child admitted to PICU with a RSV infection and requiring ventilator. In our series only half the patients who were admitted to PICU and required ventilatory support had a bacteremia revealed by PCR, meaning that antibiotic prescription might be superfluous in up to 50% of the cases. However, PCR was only performed in blood, meaning that we would be only excluding systemic bacterial infection, but not all forms of bacterial superinfection. On the other hand, due to the limited number of bacteremic subjects in our study, further studies are needed to make robust conclusions with regards to the overuse or underuse of antibiotic therapy.

In conclusion, our data show that concurrent bacteremia is not frequent in infants that are hospitalized with RSV respiratory infection, even in the presence of fever, and despite the use of molecular techniques for the diagnosis. However bacteremia may actually occur and it should be suspected in the more severe cases, although our sample size did not allow us to find a reliable predictive clinical pattern. Antibiotics are frequently overused in the setting of RSV infection, and a more judicious approach to prescription should be warranted.

## Supporting Information

S1 Fig Flow chart of the study population. (TIF)

S1 Table GENVIP Score. Clinical severity score for healthy infants with respiratory infections.
(DOCX)
