# Supplementary figures and images for: Bacteremia in Children Hospitalized with Respiratory Syncytial Virus Infection
Source: PLoS One. 2016 Feb 12;11(2):e0146599. doi: 10.1371/journal.pone.0146599 (PMC4752219; doi:10.1371/journal.pone.0146599)

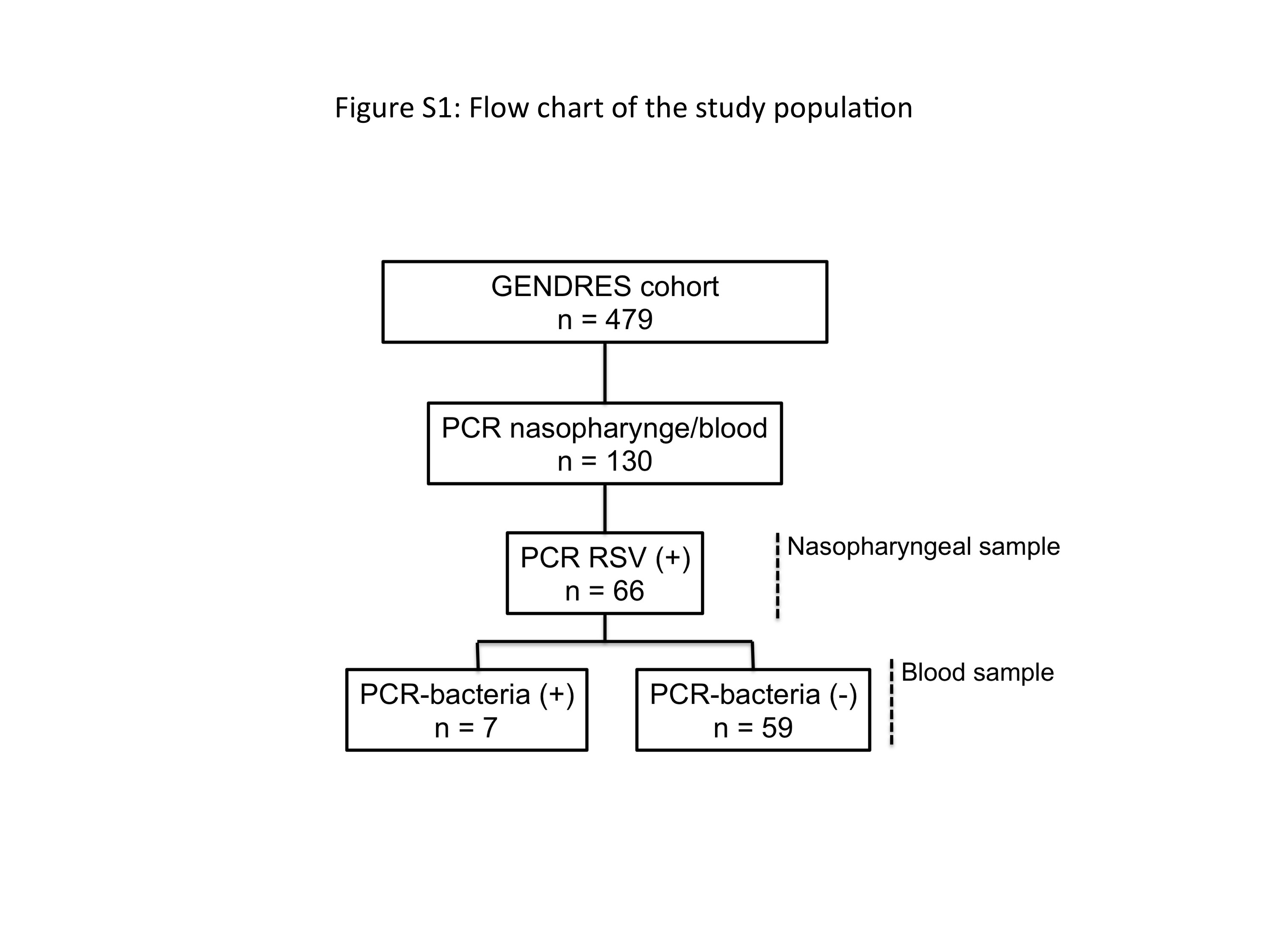

Supplement: S1 Fig — (TIF) [file pone.0146599.s001.tif]
